# Metabolite profiling of Borneo’s *Gonystylus* *bancanus* through comprehensive extraction from various polarity of solvents

**DOI:** 10.1038/s41598-023-41494-7

**Published:** 2023-09-14

**Authors:** Ika Oktavianawati, Mardi Santoso, Sri Fatmawati

**Affiliations:** 1 Department of Chemistry, Faculty of Science and Data Analytics, Institut Teknologi Sepuluh Nopember, Kampus ITS, Sukolilo, Surabaya, 60111 Indonesia; 2 Department of Chemistry, Faculty of Mathematic and Sciences, Universitas Jember, Kampus Tegalboto, Jember, 68121 Indonesia

**Keywords:** Chemistry, Chemical biology

## Abstract

*Gonystylus bancanus* wood or ramin wood has been generally known as a source of agarwood (gaharu) bouya, a kind of agarwood inferior type, or under the exported trading name of aetoxylon oil. The massive exploitation of ramin wood is causing this plant's extinction and putting it on Appendix II CITES and IUCN Red List of Threatened Species. To date, no scientific publication concerns the chemical exploration of *G. bancanus* wood and preserving this germplasm through its metabolite profiling. Therefore, research focused on chemical components profiling of *G. bancanus* is promised. This research is aimed to explore metabolomics and analyze the influence of solvent polarities on the partitioning of metabolites in *G. bancanus* wood. A range of solvents in different polarities was applied to provide comprehensive extraction of metabolites in *G. bancanus* wood. Moreover, a hydrodistillation was also carried out to extract the volatile compounds despite the non-volatile ones. LCMS and GCMS analyses were performed to identify volatile and non-volatile components in the extracts and essential oil. Multivariate data analysis was processed using Principal Component Analysis (PCA) and agglomerative hierarchical clustering. 142 metabolites were identified by LCMS analysis, while 89 metabolites were identified by GCMS analysis. Terpenoids, flavonoids, phenyl propanoids, and saccharides are some major compound classes available from LCMS data. Oxygenated sesquiterpenes, especially 10-epi-γ-eudesmol, and β-eudesmol, are the major volatile components identified from GCMS analysis. PCA of LCMS analysis demonstrated that PC1 discriminated two clusters: essential oil, dichloromethane, and *n-*hexane extracts were in the positive quadrant, while methanol and ethyl acetate extracts were in the negative quadrant. Three-dimensional analysis of GCMS data revealed that *n-*hexane extract was in the superior quadrant, and its composition can be significantly distinguished from other extracts and essential oil. *G. bancanus* wood comprises valuable metabolites, i.e., terpenoids, which benefit the essential oil industry. Comprehensive extraction by performing solvents in different polarities on *G. bancanus* wood could allow exploration of fully extracted metabolites, supported by the exhibition of identified metabolites from LCMS and GCMS analysis.

## Introduction

Peat swamp forest is a unique ecosystem with various species of vegetation^1,2^. Some commercial trees or popular timbers in Indonesia’s peatland are *Gonystylus bancanus*, *Giuta renghas* (rengas), *Combretocarpus rotundatus* (perepat) and Dipterocarpaceae from *Shorea balangeran, Shorea uliginosa,* and *Shorea parvifolia*. Valuable non-timber products also can be obtained from getah jelutung (or gum, latex) of jelutung rawa (*Dyera sp*.), gaharu or agarwood (*Aquilaria*), getah gemor (medang lendir) of *Nothaphoebe coreacea*, *Nothaphoebe cf. umbelliflora* and *Alseodaphne spp*. from Lauraceae family, bark of gemor as mosquito’s repellent, and yellowish sundi gum of *Payena leerli*^3^.

Agarwood is a commercial name for black wood containing unique dark fragrant resin formed as a response to plant defense against microbe infection in the stem, branches, and roots^4,5^. These valuable resins are produced by species from the Thymeleaceae family, i.e., *Aquilaria spp., Gonystylus spp*., *Gyrinops spp*., *Aetoxylon spp*, *Enkleia sp*, and *Wiekstroemia spp*^6–9^. Further explanation about agarwood in this article will apply the typical and local term or etymology of agarwood as gaharu, besides aloeswood, eaglewood, and oud^10^. Gaharu, particularly from *Aquilaria malaccensis*, produces the highest quality of gaharu oil, known as true-gaharu. Gaharu contains a precious resin with various medicinal uses, religious purposes, aromatic, incense, and fragrant sources^10–14^. This resin contains sesquiterpenes and phenylethyl chromones in different compositions at various plant sources^12,15,16^.

*Gonystylus spp*. and *Aetoxylon* also produce gaharu bouya (buaya) or crocodile gaharu or kemedangan, an inferior type of gaharu oil^17^, but lacks of lemon aroma given by true-gaharu^7^. Therefore, the value and price of this gaharu are lower than true-gaharu. These timbers emit specific fragrant odours depending on the type of wood. In detail, 17 species of *Gonystylus* available in Indonesia have consisted of *G. acuminatus*, *G. affinis* var. Affinis and var. elegans, *G. augescens*, *G. bancanus*, *G. borneensis*, *G. brunnescens*, *G. confuses*, *G. consanguineous*, *G. forbesii*, *G. glaucescens*, *G. keithii*, *G. macrophyllus*, *G. maingayi*, *G. micranthus*, *G. spectabilis*, *G. velutinus*, and *G. xylocarpus*^2,18–21^. In addition, two other genera in Gonystyloideae, a family of Thymelaeaceae, besides *Gonystylus* are *Aetoxylon* and *Amyxa*. These three genus are distinguished based on the morphology, shapes, and properties of each genus’s leaves and flowers^18,22^.

*Gonystylus bancanus* is endemic vegetation from Southeast Asia, especially from Borneo Island, part of Indonesia, Malaysia, and Brunei Darussalam. According to previous Indonesian vegetation data, *G. bancanus* is presented in Aceh, South Sumatera, Jambi, Riau, Bangka^18^, Central Kalimantan^23^, West Kalimantan, East Kalimantan, and South Kalimantan^24–26^. *G. bancanus* grows on ombrogen peat land in the deepest of more than 600 cm, at an elevation of 10–150 m above sea level, with soil humidity of about 72.56–84.58%, and pH of 3.4–4.0^25,27–29^. The trunk of *G. bancanus* reaches 40–45 m in height and 120 cm in diameter, with yellow sapwood in fresh logging but turning to yellowish-white after drying, and has no limit with the black heartwood part^18,24,25,28,30^. This timber has a density of 0.54–0.75 g/cm^3^ with a moisture content of 15%. The resin inside the wood is a bright yellow scented form and soft and has a lower value than *Aquilaria* resin^30^. Trade name for *G. bancanus* is commonly based on its local names, such as ramin, setalam, kayu minyak, geharu buaya (Sumatera), medang keran (West Kalimantan), merang (South Kalimantan and East Kalimantan), melawis, ramin telur, and garu buaya (Malaysia). Appendix II CITES, and IUCN Red List of Threatened Species stated that species from *Gonystylus* became critically endangered and have been conserved since 2004^25,28^. Some efforts have been conducted to preserve this genus by *ex-situ* and *in situ* conservation, such as by inventorying the population and genetic material of *Gonystylus* to manipulate the plant’s culture^31–35^.

Currently, a limited scientific paper has been discussed about *G. bancanus*^36^*.* It may be caused by the limited plant sources to be explored since it is endemic and endangered in Southeast Asian countries, especially Borneo Island. Therefore, exploring *G. bancanus* chemical profiles would be beneficial to enhance the science of tropical biodiversity, especially in Indonesia. Moreover, the prospects of its potential non-timber product, as a source of essential oil similar to aetoxylon oil, is promised. Methanol extract of *G. bancanus* wood parts, bark, sapwood, and heartwood has been evaluated for its antioxidant and antifungal activities against *Gleophyllum trabeum* and *Pycnoporus sanguineus*^37^. However, Witterseh^38^ reported that dust of *G. bancanus* wood gives allergic symptoms on respiratory and skin irritation^39–41^ since the presence of some chemicals identified by headspace GCMS inside the dust^38^.

Metabolomic techniques are practical tools for assessing the content of secondary metabolites^42^. Comprehensive metabolite analysis can be offered by the use of sophisticated technologies, including nuclear magnetic resonance (NMR), gas chromatography-mass spectrometry (GCMS), liquid chromatography-mass spectrometry (LCMS), supercritical fluid chromatography-mass spectrometry (SFCMS), direct infusion mass spectrometry (DIMS), and matrix-assisted laser desorption/ionization mass spectrometry (MALDI MS). GCMS is useful for analyzing small molecular weight metabolites with the easiness of universal mass spectral library availability. Previous research has applied GCMS as chromatography analysis on profiling secondary metabolites in plant-based food products and essential oils^43–50^. LCMS is also a favorite technique to separate and characterize semi-polar metabolites, including phenolics and terpenoids^51–55^. Combining these two methods, GCMS and LCMS, deliver comprehensive information on analyzing critical components in plants^56,57^.

The chemical nature of the samples and extraction methods mainly influences the extraction of secondary metabolites from plants. The use of solvent in the extraction step plays an essential role in recovering the chemical components inside the samples. A range polarity of solvents provides an accurate assessment of metabolite profiling on a sample containing a diverse polarity of metabolites^54,58^. Therefore, this research emphasizes the evaluation of using various polarity of solvents to extract *G. bancanus* wood.

To the best of our knowledge, this is the first article investigating metabolomic studies on ramin wood (*G. bancanus*). Total extraction using a variety of solvent polarity was applied to obtain comprehensive coverage information of metabolites in the extract of *G. bancanus* wood. Furthermore, the influence of solvent polarity on the metabolite profiling of *G. bancanus* wood was analyzed using multivariate data analysis as commonly performed by metabolomic-based research using Principal Component Analysis (PCA)^45,46,53,54,59,60^. The analysis of chemical compositions from ramin (*G. bancanus*) also may provide helpful information for future studies on the chemotaxonomy and metabolomics of *Gonystylus* to distinguish it from other species or genus in the family of Thymeleaeceae.

## Results

Sample preparation for metabolite profiling study of *G. bancanus* wood has been set up in various extraction solvents from a non-polar (*n-*hexane), semi-polar (dichloromethane), more polar (ethyl acetate), into polar solvents (methanol); and in a hydrodistillation system. The samples were extracted using a single solvent of each polarity, not a gradual fractionation of solvent polarity method. Therefore, four extracts and one essential oil variable were analyzed using LCMS and GCMS. Further data was processed using PCA, AHC, and 3D plot analysis to draw a scientific conclusion.

### Physical characteristics of the extracts and essential oil from *G. bancanus* wood

The first experiment deals with the sample extraction comprising four different solvents and hydrodistillation aimed at profiling the metabolites from ramin wood. Methanol was chosen as a solvent for wood extraction because of its advantages in extracting a vast range polarity of molecules inside the sample^61–67^. Ethyl acetate and n-hexane were also used as two solvents for extracting the wood components, as previously Kacik et al.^68^ and Yuliana et al.^67^ reported in their research on the extraction of sawdust from fir wood^68^ and *Orthosiphon stamineus* Benth^67^, respectively. Some references also recommended using dichloromethane as the extraction solvent because of its capability to extract pigments inside the wood^69–72^.

Physical characteristics, including physical appearances and the yields of these extracts, are presented in Table 1. Methanol is the best solvent for extract yield since it is commonly known for enabling polar and non-polar metabolites to interact and dissolve. The extraction procedure had no practical issue; thus, further analysis using LCMS and GCMS is projected.

**Table 1:** The yields and physical appearances of the extracts of *G. bancanus* using various solvents: *n-*hexane, dichloromethane, ethyl acetate, methanol; and water-based distillation.

Characteristics	*n-*hexane	Dichloromethane	Ethyl acetate	Methanol	Essential oil
Yield (%)	0.54 ± 0.11	3.04 ± 0.20	19.01 ± 1.89	53.71 ± 0.00	0.90 ± 0.01
physical appearance					

### Chemical components of the extracts and essential oil from *G. bancanus* wood

Data analysis of four extracts and one essential oil of ramin wood using LCMS and GCMS are presented in Tables 2 and 3, respectively. LCMS and GCMS have enabled quantifying the relative abundance of identified compounds from *G. bancanus* wood. The relative abundance was summarised considering the percentage of each identified compound calculated from the total relative abundance. This quantification style is standard and in accordance with other references discussing the quantification using GCMS^73–76^. LCMS data revealed a range of metabolites extracted adequately by a solvent in which it was soluble, while GCMS data showed a majority of terpenoids extracted from ramin wood.

**Table 2:** Metabolites identified by LCMS from *G. bancanus* extracts in n-hexane; dichloromethane; ethyl acetate; methanol, and essential oil.

No	Retention time (min)	Compounds	Relative amount (%)				
			Methanol	Ethyl acetate	Dichloro methane	*n-*hexane	Essential oil
**Flavanoid**							
**1**	7.34	3-Flavanol	1.07	1.15	1.05	0.70	1.32
**2**	9.10	3-Hydroxy-4-methoxyflavone	1.49	1.61	1.47	–	1.84
**3**	10.27	Luteolin	1.79	1.93	–	–	2.22
**4**	10.32	Kaempferol	3.17	3.41	3.11	–	3.91
**5**	10.50	Catechin	2.35	2.53	2.31	–	2.90
**6**	11.43	Quercetin	2.58	2.78	2.53	–	3.19
**7**	21.39	Vitexin	0.89	0.97	0.88	–	1.11
**8**	22.62	Kaempferol-3-O-D-glucoside	1.24	1.34	–	–	–
**9**	22.63	Luteolin-7-glucoside	0.93	1.01	–	–	–
**10**	25.85	Isorhamnetin–3-O-b-D-galactopryranoside	1.24	1.34	–	–	–
**11**	30.87	Kaempferol-3-(6"-malonylglucoside)	2.54	2.73	–	–	–
**12**	31.82	Quercetin-3-O-malonylglucoside	2.82	3.04	–	–	–
**13**	33.43	Procyanidin A1	2.21	2.38	–	–	–
**14**	33.43	Procyanidin A2	2.63	2.84	–	–	–
**15**	33.50	Procyanidin B1	2.03	2.19	–	–	–
**16**	33.50	Procyanidin B2	1.77	1.91	–	–	–
**17**	35.51	Quercetin-3-glucoside-7-rhamnoside	3.05	3.29	–	–	–
**18**	35.52	Rutin	2.43	2.62	–	–	–
***Total flavonoids***			**36.25**	**39.06**	**11.35**	**0.70**	**16.49**
**Phenyl propanoid**							
**19**	1.58	Cinnamic acid	1.07	1.16	–	–	1.32
**20**	2.65	Safrole	–	0.56	1.39	1.70	1.51
**21**	2.65	*p*-Coumaric acid	0.61	0.65	–	–	–
**22**	4.64	Caffeic acid	2.08	2.24	–	–	2.57
**23**	5.04	Ferulic acid	1.24	1.34	–	–	1.53
**24**	7.03	Sinapic acid	0.65	0.71	–	–	–
**25**	11.62	3-*p*-Coumaryl-1,5-quinolactone	1.87	2.02	1.84	–	1.22
**26**	11.63	3-Caffeoyl-1,5-quinolactone	1.11	1.19	1.09	–	–
**27**	12.05	*p*-Coumaroylquinic acid	0.98	1.06	0.97	–	–
**28**	12.15	3-Feruloyl-1,5-quinolactone	0.81	0.87	0.79	–	–
**29**	12.42	Chlorogenic acid	1.78	1.92	–	–	2.20
**30**	12.44	Quinic acid-4-O-caffeate	1.24	1.34	1.22	–	–
**31**	12.72	3-O-Feruloylquinic acid	2.16	2.33	2.13	–	2.67
***Total phenyl propanoids***			**15.62**	**17.39**	**9.42**	**1.70**	**13.03**
**Terpenoid**							
**32**	1.03	Isoprene	–	0.13	0.31	0.38	0.34
**33**	1.47	*p*-Cymene	–	0.13	0.32	0.39	0.35
**34**	1.48	α-Terpinene	–	–	1.19	1.45	1.29
**35**	1.48	α-Pinene	–	–	1.73	2.12	0.68
**36**	1.48	b-Pinene	–	0.48	–	1.44	1.29
**37**	1.48	Sabinene	–	0.64	1.58	1.93	1.72
**38**	1.49	Limonene	–	1.32	3.28	4.00	3.57
**39**	1.49	α-Terpinolene	–	0.33	0.82	1.00	1.02
**40**	1.50	b-Phellandrene	–	0.99	2.45	3.00	2.67
**41**	1.50	b-Myrcene	–	0.71	1.76	2.15	1.92
**42**	1.52	g-Terpinene	–	0.76	–	2.31	2.06
**43**	1.64	Linalool	–	1.37	3.39	4.14	3.70
**44**	1.65	Isoborneol	–	0.85	2.10	2.56	2.29
**45**	1.66	cis-Rose oxide	–	1.04	2.57	3.14	2.80
**46**	1.71	Geranial	–	–	–	–	1.34
**47**	1.76	*p*-Menthadiene	–	0.41	1.00	1.23	1.10
**48**	1.79	Isomenthol	–	1.45	3.58	4.38	3.90
**49**	1.80	Neoisomenthol	–	–	0.97	1.19	1.15
**50**	2.82	a-Calcorene	–	0.15	–	0.44	–
**51**	5.48	α-Cubebene	–	0.33	–	1.00	0.56
**52**	5.49	α-Copaene	–	0.61	1.50	1.84	1.64
**53**	5.49	Longifolene	–	0.61	1.50	1.84	–
**54**	5.50	b-Chamigrene	–	0.15	0.37	0.46	0.41
**55**	5.50	b-Selinene	–	0.43	1.08	1.32	1.18
**56**	5.50	b -Bisabolene	–	0.13	0.32	0.39	0.35
**57**	5.50	b -Elemene	–	0.21	0.52	0.63	0.65
**58**	5.51	a-Humulene	–	0.25	0.62	0.76	0.68
**59**	5.51	a -Muurolene	–	–	–	0.44	0.39
**60**	5.52	(E,E)-a-Farnesene	–	0.49	1.20	1.47	1.31
**61**	5.53	a-Bourbonene	–	0.94	2.33	2.84	2.54
**62**	5.54	Allo-aromadendrene	–	0.72	1.78	2.18	1.94
**63**	5.55	a-Cadinene	–	0.65	1.61	1.96	1.75
**64**	5.55	a-Ylangene	–	0.49	1.20	1.47	0.87
**65**	5.56	Bicyclo-germacrene	–	0.41	1.00	1.23	1.53
**66**	5.57	g-Cadinene	–	0.30	0.75	0.92	0.82
**67**	5.60	g-Muurolene	–	0.47	1.18	1.44	1.28
**68**	5.99	Selina-4,11-diene	–	0.63	1.55	1.89	1.69
**69**	6.41	11-Selina-4-a-ol	–	–	0.52	0.63	0.56
**70**	6.95	τ-Cadinol	–	0.28	0.70	0.86	–
**71**	6.95	Nerolidol	–	–	0.95	1.16	1.03
**72**	6.97	Farnesol	–	0.49	1.20	1.47	1.31
**73**	6.97	10-Epi-g-eudesmol	–	0.46	1.13	1.38	1.23
**74**	6.97	b-Eudesmol	–	0.32	0.78	0.96	0.86
**75**	6.98	Elemol	–	0.26	0.64	0.78	0.31
**76**	9.35	9b-Pimara-7,15-diene	–	0.32	0.79	0.97	0.86
**77**	9.36	Ent-sandaracopimaradiene	–	0.21	0.51	0.63	–
**78**	9.37	Stemar-13-ene	–	0.76	1.89	2.31	2.06
**79**	9.73	Ent-cassa-12,15-diene	–	–	0.69	0.85	0.76
**80**	17.05	d7-avenasterol	0.42	0.45	0.41	0.27	–
***Total terpenoids***			**0.42**	**22.09**	**55.78**	**73.62**	**61.74**
**Alkaloid**							
**81**	1.49	Trigonelline	0.46	0.49	–	–	–
**82**	1.49	3-Ethyl-2,5-dimethylpyrazine	0.11	0.12	0.11	0.07	–
**83**	1.72	Pipecolic acid	0.12	0.13	–	–	–
**84**	1.72	2-Ethenyl-3,5-dimethylpyrazine	0.25	0.28	0.25	0.17	–
**85**	1.76	3-Isopropyl-2-methoxypyrazine	0.09	0.10	0.09	0.06	–
**86**	2.55	Xanthine	0.11	0.12	0.11	0.07	0.07
**87**	2.80	7-Methylxanthine	0.54	0.58	0.53	0.36	–
**88**	2.80	3-Methylxanthine	1.11	1.19	1.09	0.73	0.05
***Total alkaloids***			**2.80**	**3.02**	**2.19**	**1.47**	**0.12**
**Furanoids**							
**89**	1.21	Furfural	0.65	–	–	–	–
**90**	1.26	2-Furfurylthiol	0.29	0.32	0.29	2.00	–
**91**	1.46	2,5-Dimethyl-4-hydroxy-3(2H)-furanone	0.33	0.35	0.32	–	–
**92**	1.71	3-Hydroxy-4,5-dimethylfuran-2(5H)-one	0.22	0.24	0.21	–	–
**93**	2.23	(-)-5-Ethyl-3-hydroxy-4-methyl-2(5H)-furanone	0.26	0.28	0.26	–	–
**94**	2.24	2-Ethyl-4-hydroxy-5-methylfuran-3-one	0.32	0.35	0.32	–	–
***Total furanoids***			**2.08**	**1.53**	**1.39**	**0.20**	**0.00000**
**Saccharides**							
**95**	1.60	Arabinose	1.24	–	–	–	–
**96**	1.84	Rhamnose	1.61	–	–	–	–
**97**	2.54	Xylose	1.65	–	–	–	–
**98**	12.14	Inulobiose	2.17	–	–	–	–
**99**	12.96	3-Methylbutanoyl-1-O-b-D-glucopyranosyl-b-D-apiofuranoside	1.11	1.19	–	–	–
**100**	26.30	1-Kestose	1.64	–	–	–	–
**101**	26.31	Raffinose	1.24	–	–	–	–
**102**	26.31	Inulotriose	2.78	–	–	–	–
**103**	43.21	Inulotetraose	2.80	–	–	–	–
**104**	43.21	Nystose	2.49	–	–	–	–
**105**	46.17	Mannan	3.61	–	–	–	–
**106**	46.18	Stachyose	3.30	–	–	–	–
**107**	46.18	1(F)-a-D-galactosylraffinose	3.28	–	–	–	–
**108**	49.95	1F-fructofuranosylnystose	3.26	–	–	–	–
**109**	54.18	Glucomannan	2.42	–	–	–	–
***Total saccharides***			**34.61**	**1.20**	**0.00000**	**0.00000**	**0.00000**
**Aromatic**-**Benzene**							
**110**	1.23	Benzaldehyde	0.27	0.29	0.26	0.18	–
**111**	1.29	Benzoic acid	0.46	0.49	–	–	0.56
**112**	1.30	2-Phenylethyl alcohol	–	0.62	1.54	1.88	–
**113**	1.71	2-Methoxy-4-vinylphenol	0.33	0.36	0.32	0.22	–
**114**	1.71	2,6-Dimethyl-decahydronaphthalene	–	0.11	0.29	0.35	–
**115**	1.76	4'-Ethylacetophenone	–	0.13	0.31	0.38	–
**116**	1.76	4'-Methoxy acetophenone	–	0.17	0.41	0.51	–
**117**	5.03	Isopentyl benzoate	0.46	0.49	0.45	0.30	–
***Total aromatics***			**1.51**	**2.66**	**3.59**	**3.82**	**0.56**
**Miscellaneous**							
**118**	0.96	Methyl formate	–	0.56	1.39	1.71	–
**119**	0.96	1,3-pentadiene	–	0.26	0.64	0.78	0.70
**120**	1.17	2,3-Butanedione	0.35	0.37	0.34	0.23	–
**121**	1.20	2-Methylhexane	–	0.17	0.43	0.52	0.46
**122**	1.20	Methyl butyrate	–	0.41	1.01	1.23	–
**123**	1.21	Valeric acid	0.42	0.46	–	–	–
**124**	1.22	3-Hexanone	0.22	0.25	0.21	–	–
**125**	1.24	4-Heptenal	0.26	0.28	0.26	0.17	0.07
**126**	1.25	Succinic acid	0.18	0.19	–	–	–
**127**	1.29	Methyl 3-methylbutanoate	0.11	0.12	0.11	0.07	–
**128**	1.43	1,5-Octadien-3-one	0.39	0.43	0.39	0.26	0.21
**129**	1.46	Isopropyl butyrate	0.33	0.35	0.32	–	–
**130**	1.49	1-Octen-3-ol	–	0.41	1.00	1.23	1.10
**131**	1.52	1-Octen-3-one	0.26	0.28	0.26	0.17	0.06
**132**	1.54	a-ketoglutaric acid	0.33	0.35	–	–	0.40
**133**	1.56	3-Hexenyl acetate	–	1.07	2.64	3.23	–
**134**	1.72	(2R)-Nonan-2-ol	–	1.13	2.79	3.42	–
**135**	1.72	1-Butyl-2-propylcyclopentane	–	0.26	0.64	0.78	0.70
**136**	2.82	1-Octen-3-yl acetate	–	0.30	0.75	0.91	–
**137**	4.65	2,2,6-Trimethyldecane	–	0.45	1.12	1.37	–
**138**	4.65	4,6-Dimethylundecane	–	0.27	0.67	0.82	–
**139**	5.02	Quinic acid	0.61	0.65	–	–	–
**140**	7.34	2-Hexyl-1-decanol	–	0.52	1.30	1.59	1.41
***Total miscellaneous***			**3.46**	**9.56**	**16.27**	**18.50**	**5.100**
**141**	3.04	Gallic acid	**2.39**	**2.58**	–	–	**2.96**
**142**	5.18	Syringic acid	**0.85**	**0.91**	–	–	–
**Total**			**100.00**	**100.02**	**100.00**	**100.00**	**100.00**

Significant values are in bold.

**Table 3:** Metabolites identified by GCMS from *G. bancanus* extracts in *n-*hexane; dichloromethane; ethyl acetate; methanol, and *G. bancanus* essential oil.

No	Retention time (min)	Compounds	Relative amount (%)				
			Methanol	Ethyl acetate	Dichloro methane	*n-*hexane	Essential oils
	Oxygenated monoterpenes						
1	28.13	Myrcenyl acetate	–	–	–	–	0.31
2	28.76	Dihydrocarvyl acetate	2.63	2.12	2.57	4.13	3.13
3	29.05	a-Limonene diepoxide		–	–	0.44	–
4	29.82	Myrcenol		–	–	–	0.11
5	29.95	Linalool	1.69	–	–	–	–
6	31.82	Neryl acetone	0.26	–	0.99	–	–
7	32.13	4-Methyl isopulegone	–	2.15	–	–	–
8	32.20	a-Pinene epoxide	–	–	0.95	0.48	–
9	32.44	Myrtenal	–	–	1.39	–	–
10	33.19	Limonene dioxide 1	3.86	3.71	2.94	0.53	–
11	33.63	Eucalyptol	–	0.61	–	–	–
12	33.64	cis-Myrtanol	–	–	–	0.28	–
13	34.13	1-Methyl-4-(2-methyloxiranyl) -7-oxabicyclo 4.1.0 heptane	7.82	5.02	7.56		–
14	36.04	a-terpineol	–	2.31	2.65		1.38
15	37.69	a-terpinyl acetate	0.95	0.73	0.98	1.06	–
16	37.77	(E)-5-Isopropyl-6,7-epoxy-8-hydroxy-8-methylnon-2-one	–	–	0.70		–
17	37.96	Perilic alcohol	–	–	0.45		–
18	39.48	Isothujol	–	0.68	–		–
19	61.10	3-(Acetylmethyl)-b-pinene	–	–	–	0.35	–
		**Total oxygenated monoterpenes**	**17**.**21**	**17**.**33**	**21**.**18**	**7**.**27**	**4**.**93**
	**Hydrocarbon Sesquiterpenes**				–		
20	25.35	a-humulene	1.18	0.92	0.49	0.44	0.39
21	25.35	Junipene	–	–	–	0.76	
22	25.71	a-gurjunene	0.32	–	–	–	–
23	25.84	Ledane	–	0.70	–	–	–
24	26.02	b-Caryophyllene	0.60	0.62	–	0.57	0.22
25	26.50	a-Copaene	–	–	–	0.63	
26	28.78	b-eudesmene	–	–	–	–	0.28
27	31.57	Farnesene	–	1.40	–		0.16
28	35.04	Patchoulane	–	–	–	0.32	0.30
29	35.05	b-Elemene	–	–	0.70	–	0.19
30	38.79	3,3,6,6,9,9-Hexamethyl-tetracyclo[6.1.0.0(2,4).0(5,7)] nonane	–	4.59	3.22	–	–
		**Total hydrocarbon sesquiterpenes**	**2**.**1**	**8**.**23**	**4**.**41**	**2**.**72**	**1**.**54**
	**Oxygenated Sesquiterpenes**						
31	25.84	b-Caryophyllene epoxide	–	–	0.64	0.75	
32	25.86	Farnesol	0.59	–	–	–	0.16
33	27.38	Elemol	–	1.46	1.14	2.38	
34	27.56	Nerolidol	0.48	0.95	0.85	1.70	0.54
35	28.97	Ledol	–	–	0.46	–	–
36	29.73	10-epi-γ-eudesmol	36.21	26.54	27.28	26.95	60.48
37	29.89	Veridiflorol	–	1.58	–	–	–
38	30.26	b-Eudesmol	31.41	22.47	19.17	19.25	28.04
39	32.56	Endo-8-hydroxy-cycloisolongifolene	–	0.71	–	–	–
40	35.51	a-Bisabolol	–	3.12	–	–	–
41	35.59	a-Copaene-11-ol	–	–	3.56	–	–
42	36.20	Longipinenepoxide	0.65	–	–	0.57	0.55
43	36.44	a-Bisabololoxide-B	–	1.2	1.44	–	–
44	61.61	Spathulenol	–	–	–	0.28	–
45	65.55	Nerolidol	–	–	0.94	3.46	–
46	69.15	Globulol	–	–	–	0.41	–
		**Total oxygenated sesquiterpenes**	**69**.**34**	**58**.**03**	**55**.**48**	**55**.**75**	**89**.**77**
	**Oxygenated Diterpenes**						
47	38.28	17-Acetoxy-19-kauranal	–	–	0.87	–	–
48	54.04	Geranyl linalool	–	–	0.50	0.48	–
49	62.99	Kauren-19-yl-acetate	–	–	–	0.49	–
50	67.75	Kauren-18-ol acetate	–	–	–	2.11	–
		**Total oxygenated diterpenes**	–	–	**1**.**37**	**3**.**08**	–
	**Hydrocarbon Triterpenes**				–		
51	51.60	Squalene		1.81	1.79	7.17	
		**Total hydrocarbon triterpenes**	–	**1**.**81**	**1**.**79**	**7**.**17**	–
	**Oxygenated Triterpenes**						
52	68.08	Dicholesteryl succinate	–	–	–	0.62	–
53	68.49	4-Methyl-(3b,4a)-cholesta-8,24-dien-3-ol	–	–	0.94	1.09	–
		**Total oxygenated triterpenes**	–	–	0.94	1.71	–
	**Miscelaneous**						
54	27.41	Methyl arachidonate	0.75	–	–	–	–
55	27.43	Methyl heptadec-trans-10-en-8-ynoate	0.67	–	–	–	
56	27.73	Caprylic acid	–	–	–	–	0.18
57	30.56	6-Methyl-5-heptene-2-one	5.08	2.72	2.20	1.34	2.05
58	31.28	9,12,15-Octadecatrienal	–	–	–	–	0.69
59	31.44	2-Undecyl cyclopropanepentanoic acid methyl ester	0.24	–	–	–	–
60	32.01	7-Hydroxymethyl-8-ethoxy-cis-bicyclo(4.3.0)-3-nonene	–	0.91	–	–	–
61	32.14	2-Pentadecyn-1-ol	0.36	–	–	–	–
62	33.65	Trans-2-undecenal	0.52	–	–	–	–
63	33.66	Methyl 5,8-octadecadienoate	–	–	1.53	–	–
64	34.04	Allyl heptanoate	–	–	–	0.33	–
65	35.16	2,5-Dimethyl-2,5-hexanediol	–	–	0.64	–	–
66	35.82	3,4-dimethyl-3-penten-2-one	–	–	–	–	0.08
67	35.91	Methyl 14-methyl pentadecanoate	1.01	–	–	0.71	–
68	36.66	Butyl phthalate	–	2.17	–	–	–
69	36.73	9,12,15-Octadecatrienal	–	–	–	–	0.75
70	36.78	Stearic acid	–	–	2.11	3.66	–
71	39.49	Methyl 11-octadecenoate	1.38	–	–	1.02	–
72	39.61	Methyl 15-tetracosenoate	0.45	–	–	–	–
73	39.98	Methyl stearate	0.89	–	–	–	–
74	40.41	Oleic acid	–	4.43	4.37	13.35	–
75	40.90	Isopropyl myristate	–	–	–	0.78	–
76	48.73	Methyl 9,12,15-octadecatrienoate	–	–	–	0.44	–
77	48.84	Jasmonol	–	–	–	0.25	–
78	50.58	4-Butyl phenol	–	3.06	2.9	–	–
79	56.86	4-Methoxymethyl phenol	–	–	0.46	–	–
80	58.81	Carinol	–	1.32	–	–	–
		**Total miscelaneous**	**11**.**35**	**14**.**61**	**14**.**21**	**21**.**88**	**3**.**75**
		**Total**	**100.00**	**100.01**	**99.38**	**99.58**	**99.99**

(–) = not detected.

Significant values are in bold.

It can be observed from the presented data in Table 2 that LCMS could identify not only non-volatile components but also the volatile ones in ramin wood, such as terpenoids, while GCMS in Table 3 mostly captured and detected the volatile compounds. This compiled extraction and chromatography analysis allows a comprehensive coverage of the ramin wood metabolome by partitioning metabolites between different extraction fractions and by the wide-ranging polarity of compound identifications.

### Statistical analysis

**Figure 1:**
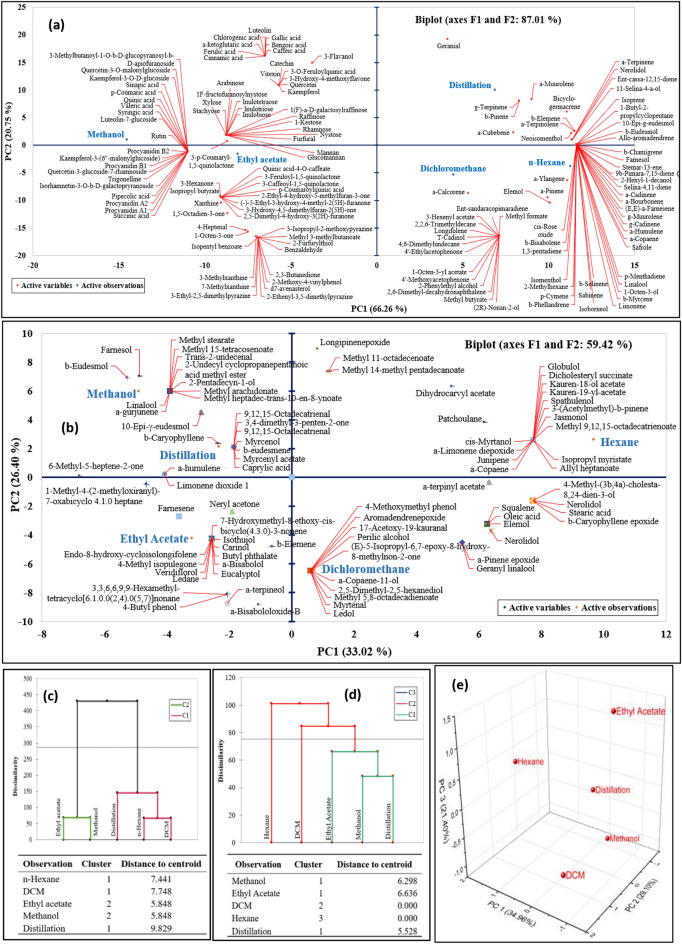
Principal Component Analysis (PCA) biplot of extracts and essential oil from *G. bancanus* based on LCMS analysis (**a**) and GCMS analysis (**b**). The variable descriptions were referred to as their corresponding compounds in Tables 2 and 3. Hierarchical Clustering Analysis (HCA) dendrogram of the current data analysis for LCMS resulting in two data clusters (**c**) and GCMS resulting in three data clusters (**d**) of the extracts and essential oil from *G. bancanus*. Since the eigenvalue cumulative of PC1 and PC2 in GCMS statistical analysis was less than 80%, then 3D analysis of it was determined using Origin software (**e**).

Data mining was processed through PCA, AHC, and 3D plot analysis, as shown in Fig. 1. This multivariate data analysis permits the correlation of the metabolite profile obtained from different extraction solvents. The metabolites identified as Principal Components (PCs) were considered a variable, and each metabolite's score or relative abundance was calculated. The biplot of the first two PCs in Fig. 1a explained that 87.01% of the total variance was sufficient to represent data for analysis. PC1 separated the metabolic profiles into two clusters: (1) *n-*hexane extract, dichloromethane extract, and essential oil were on the positive side, while (2) ethyl acetate and methanol extracts were on the opposing side. This analysis was supported by AHC visualization of LCMS data, which revealed a distinct chemical relationship between these two extract clusters, as shown in Fig. 1c.

As observed in Fig. 1b, data sets PC1 strongly discriminated chemical components of *n-*hexane extract from other extracts and essential oil. However, the PCA of the first two PCs was not representative in distinguishing chemical composition among extracts since the total variance of the data set was less than 80%, i.e., 59.42%. Eigenvalue PC2 of 59.42% required more matrix dimension to explain demonstrative information, thereby increasing variance into the third component (PC3) was compulsory. Figure 1e shows that the score plot of the first three principal components explains 85.46% of the total variance. This three-dimension matrix of PCA supported the conclusion of clustering analysis, AHC, in Fig. 1d. PC2 separated two clusters: dichloromethane and *n-*hexane in the superior quadrant, while the rest of the extracts (ethyl acetate and methanol) and essential oil in the inferior quadrant. Therefore, Fig. 1d exhibited three clusters consisting of (1) *n-*hexane extract; (2) dichloromethane extract; and (3) essential oil, ethyl acetate, and methanol extracts.

## Discussion

*G. bancanus* wood or ramin wood as a source of essential oil from inferior agarwood type has attracted attention from many business industries, especially the flavor and fragrance industry. The study on the exploration of ramin wood phytochemicals is less known. Many researchers, mainly from Indonesia, focused on the population inventory of ramin wood and its bioactivity assessment. Therefore, based on LCMS and GCMS data, this article is worth discussing the structural diversity within the annotated compound classes in *G. bancanus* wood.

**Figure 2:**
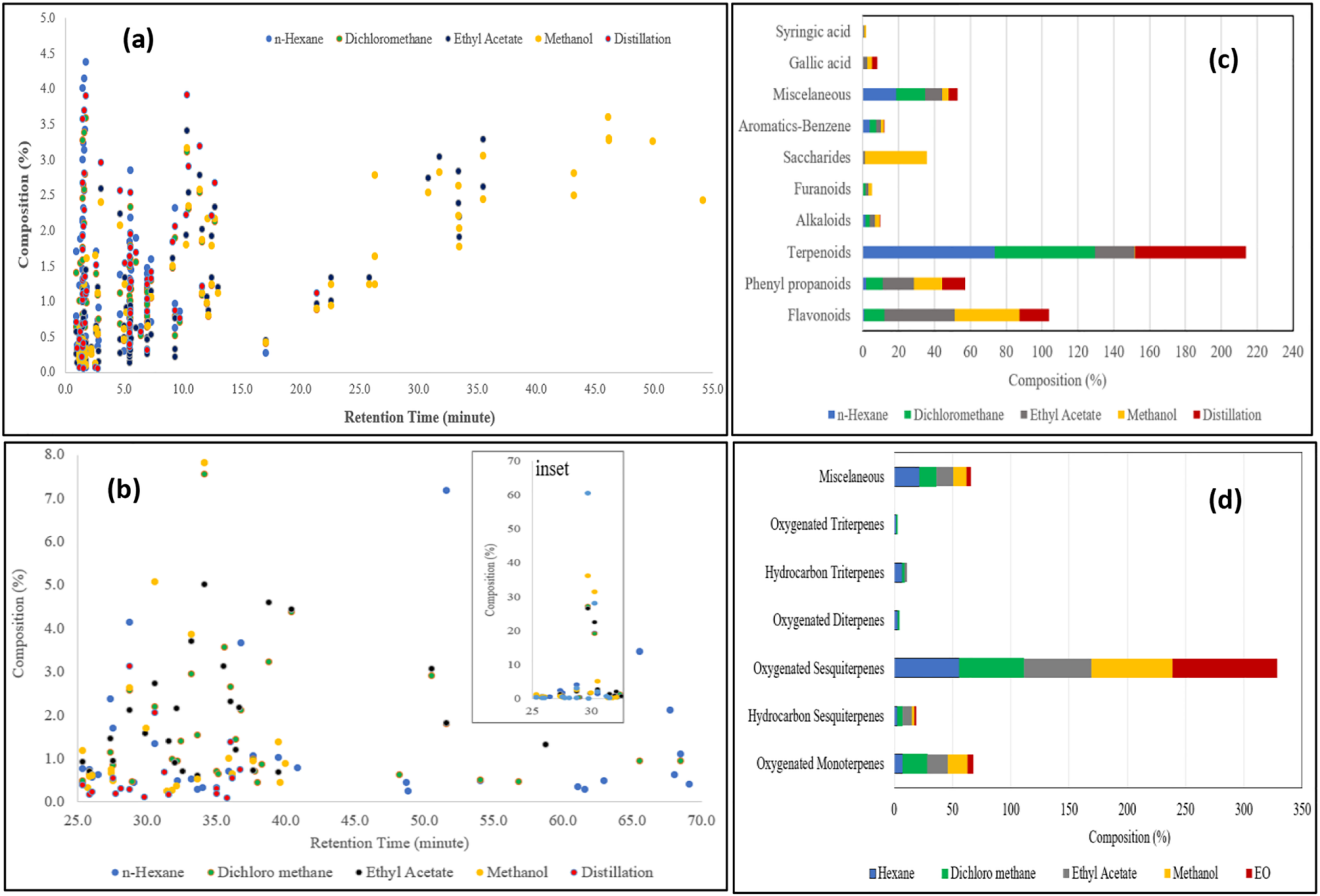
Distribution of retention time and relative amount of current metabolites on LCMS (**A**), and GCMS analysis (**B**) from the extracts and essential oil of *G. bancanus*. The dots in blue refers to the identified compounds in *n-*hexane extract. In contrast, the green, black, yellow and red refers to the identified compounds in dichloromethane, ethyl acetate, methanol and essential oil obtained from distillation, respectively. Clustering of the extracts and essential oil metabolites were performed in clustered bars based on LCMS (**C**) and GCMS (**D**) data in Tables 2 and 3.

Non-targeted analysis of *G. bancanus* metabolite profiles was obtained from solvent-varied extracts and essential oil and a combination of LCMS and GCMS chromatography analysis. The chromatographic and mass spectral data were interpreted for 142 compounds from LCMS and 89 compounds from GCMS. Both data analysis is presented in Tables 2 and 3. In comparison, the distribution of their retention times and relative amount, sorted by compound class, is shown in Fig. 2. The influence of solvent polarity on metabolite profiling is also discussed based on multivariate data analysis.

### Physical characteristics of the extracts and essential oil from *G. bancanus* wood

As shown in Table 1, the extractives performed a range of colors mainly affected by the chemical content extracted from the wood using current solvents. The darker extracts may represent highly concentrated components, particularly coloring matters. Coloring matters in plants, commonly known as pigments, comprise flavonoids, polyphenols, tannins, and carotenoids^77^. The degree of solvent polarity used in this research has influenced the order of the darkness of extracts. Methanolic extract showed the darkest extract among others.

Methanol has been known as a universal solvent to extract a broad range of polarity of compounds. The data from LCMS analysis in this research has supported this statement. Methanolic extracts contain a bulky polar metabolite from flavonoid and phenyl propanoid groups. Flavan-3-ol, as the building block of natural dimers of proanthocyanidins and polymers of tannins in plants^77^, was also found with its derivatives inside this methanolic extract. In addition, many forms of saccharides were also presented in considerable amounts in the methanolic extracts, which may influence its turbidity.

Methanol has a considerable chemical potency for extracting the polar metabolites since it is a light, volatile liquid, and protic solvent more likely to interact with polar components in the wood, such as tannins, polar polymers, and saccharides. It also fully redissolves dried polar extractives inside the wood. Methanol is less dense than water which accelerates the motion or diffusion of a solvent through the wood cells. In addition, Malik et al. stated that secondary metabolites from plants would disperse in methanol and lead methanol molecules to escape from the bonds quickly after collecting enough kinetic energy from its exchange with neighbor molecules. It resulted in methanol quickly leaving the mass of liquids to join the air as a vapor^78^. Therefore, methanol is a good option for choosing an organic solvent to extract wood matters in flavonoid, phenolic, furanoid, and saccharide components.

However, the methanolic extract of *G. bancanus* wood contained significantly fewer terpenoids. These terpenoids are mostly volatile compounds and non-polar components with various hydrophobic groups and van der Walls interactions. These compounds are more soluble in non-polar solvents like n-hexane than in methanol. Therefore, terpenoids were extracted mainly in n-hexane and semi-polar solvents like dichloromethane and ethyl acetate.

Ethyl acetate followed the behavior of methanol in extracting chemical components inside the wood. It also extracted many flavonoids and phenyl propanoids, but significantly less for saccharides. Therefore, ethyl acetate extract appeared dark but less than methanolic extract. This phenomenon decreased with the increasing order of non-polarity of extraction solvents. The n-hexane extract has the lightest color extract compared to other extractives. It was claimed to extract most terpenoids in the wood sample effectively. Previous research by Robinson et al. mentioned that dichloromethane was the best solvent for extracting pigments compared to some selected solvents, including water^70,71^. However, when dichloromethane was compared to methanol for extracting the wood components in this research, it showed a weaker interaction during extraction and solubilization of the pigmented matters in *G. bancanus*.

### Chemical components in *G. bancanus* wood

A total of 142 metabolites were annotated from LCMS analysis in Table 2. The distribution of their retention times and relative amount sorted by compound class is shown in Fig. 2. This separation is based on the compound's interaction between the solutes and mobile phase and the column of LC. The results indicated that non-polar metabolites in methanol extract are eluted at the end of liquid chromatographic separation, especially for long-chain carbon, branched saccharides, and glucomannan. Numerous primary metabolites of saccharides can be detected from *G. bancanus* wood in significant amounts, approximately 34.61% of total compounds, while the rest are secondary metabolites.

The identified saccharides in *G. bancanus* wood mainly comprise fructose- and sucrose-derived compounds. Fructose-derived compounds contain their parent compound, i.e., fructans, polysaccharides of fructose linked by β-(2→1) glycosidic bond, and are generally known as prebiotic sources for dietary supplements and diabetic-suitable sweeteners. Fructans accumulate in the cell vacuoles and act as carbon sinks within the cell to facilitate photosynthesis. Inulin, a part of fructans, is also presented in the form of its hydrolyzed forms in plants, including inulobiose, inulotriose, and inulotetraose. Whenever inulin is available in plants to store energy, plants do not keep any other form of carbohydrate, such as starch^79,80^. Stachyose and 1(F)-α-D-galactosylraffinose are derivatives of raffinose, a galactosyl substituted sucrose-derived compound. These three compounds are presented in ramin wood. Fructooligosaccharides (FOS), another sucrose-derived prebiotic, were found in the methanolic extract as 1-kestose, nystose, and 1F-fructofuranosylnystose.

Other forms of saccharides in ramin wood are monosaccharides, including xylose, arabinose, and rhamnose, and a polysaccharide of mannose, mannan, and its derivative, glucomannan. The least detected saccharide in the methanolic extract of ramin wood is 3-methylbutanoyl-1-O-β-D-glucopyranosyl-β-D-apiofuranoside. This compound is a sugar ester in the form of acyl disaccharide, commonly found as the bound flavour constituent in green arabica coffee beans^81^.

Flavonoids in *G. bancanus* wood comprise a wide range of subclasses, such as flavones, flavonols, flavanols, and flavanonols. Flavones, with a backbone of 2-phenylchromen-4-one, consisted of kaempferol, quercetin, and their glycoside derivatives and comprised half of the flavonoids in ramin wood extracts of methanol and ethyl acetate. Flavanols are the second major compound group found in ramin wood including 3-flavanol, catechin and procyanidin-based compounds such as procyanidin A1, procyanidin A2, procyanidin B1 and procyanidin B2. Apigenin glycoside (vitexin), luteolin and its glycoside were three compounds of flavonols, while 3-hydroxy-4-methoxyflavone, and isorhamnetin-3-O-β-D-galactopyrranoside were two compounds of flavanonols found in ramin wood. Previous research of those flavonoid compounds also presented in ramin wood showed generous bioactivities as an antioxidant, antidiabetic, antimicrobe, etc^82–85^.

Previous research by Ahmad^86^ showed that a yellow pigment of 5-hydroxy-7,4′-dimethoxyflavone has been isolated from the heartwood of *G. bancanus*. This flavone has been investigated to exhibit antimicrobial activity against *Candida albicans*^87^, *Staphylococcus aureus, Proteus vulgaris, Escherichia coli*^88^; anti-allergic action against antigen-induced β-hexosaminidase^89^; cytotoxic effect in lines of colon cancer (RKO) and cerebral astrocytoma (D-384)^90^; hypolipidemic effect in vivo experiments^91^ and insulinotropic effect^92^. However, this flavone is toxic in human lymphocytes^93^. Remarkably, based on our research result, no signal was found for 5-hydroxy-7,4′-dimethoxyflavone, but other flavones, such as luteolin and vitexin, were presented. Agarwood from *Aquilaria* genus also produced some apigenine and luteolin glycosides^94^.

The phenylpropanoid backbone-based compounds found in ramin wood were cinnamic acid having a honey-like odour; hydroxycinnamic acids consisting of p-coumaric acid, caffeic acid, ferulic acid, and sinapic acid; and hydroxybenzoic acid in the form of gallic acid (3,4,5-trihydroxybenzoic acid). The presence of hydroxycinnamic acids in the wood may influence the wood’s colour, enhancing and stabilising the pigment. Other phenylpropanoids in ramin wood extract were cinnamic acid esters, including 3-p-coumaroyl-1,5-quinolactone; 3-caffeoyl-1,5-quinolactone; and 3-feruloyl-1,5-quinolactone. Chlorogenic acids as a polyphenol family of hydroxycinnamic acid esters were presented as p-coumaroylquinic acid (pCoQA), 4-O-caffeoylquinic acid (4-CQA), and 3-O-feruloylquinic acid (3-FQA). Safrole, previously known to have a sweet- and candy-shop aroma as a food flavor, and currently known as the precursor of drug synthesis of ecstasy 52–54, was also found in *G. bancanus* wood. Those phenylpropanoid derivatives have been reported to serve as aroma and flavoring agents and have a wide range of bioactivities such as antifeedant, insecticide synergist, antibiotic, and anti-angiogenic^95–99^.

Terpenoids were presented in abundant amounts from ramin wood extracts and divided into subclasses, including monoterpenes, sesquiterpenes, and triterpenes. Terpenoids could be detected using LCMS and GCMS analysis. In this research, 49 terpenes were reported from LCMS analysis, while GCMS also detected 53 terpenes. Terpenes in hardwood are primarily found in the leaves and the resin inside sapwood. The presence of terpenes in the resin may reduce the resin’s viscosity, thus flowing the resin into a damaged part of the tree and creating a hydrophobic cover to protect the tree from further damage. Generally, the volatile mono- and sesquiterpenes, and aromatics are emitted into the air by a tree as allelochemicals to defend against herbivory. It also acts as a warning to herbivores that the current plant is no longer edible and as an alert to the natural enemy of the presence of the plant invaders. The non-volatile diterpenes acting as phytoalexins against microbial infection are left in the resin wood^100–104^.

The research indicated that *G. bancanus* wood contained isomenthol, linalool, limonene, 1-methyl-4-(2-methyloxiranyl)-7-oxabicyclo 4.1.0 heptane, and limonene dioxide as dominant monoterpenes. While 10-epi-γ-eudesmol, β-eudesmol, α-bourbonene, allo-aromadendrene, and selina-4,11-diene are the dominant one from sesquiterpenes. Polycyclic diterpenes are often attractive since their defense role is to protect and recover the plant from disease caused by fungi and bacteria. Tricyclic diterpenes, including 9β-pimara-7,15-diene; ent-sandaracopimara-8(14),15-diene; and ent-cassa-12,15-diene are common precursors of phytoalexins that are existed in ramin wood. Furthermore, tetracyclic diterpenes have skeletons of stemarane and kaurene, and sterol-based triterpenes such as (7-avenasterol may play an important role in plant growth hormone are also found in this research.

A range of alkaloids was fully extracted from ramin wood, with xanthine and its derivatives as the major ones. Trigonelline, a polar hydrophilic alkaloid, which has been reported in higher concentrations in seeds of legumes and coffee, was also extracted from ramin wood. Pyrazine-based compounds in this ramin wood have resulted in a nutty roasted odor, a fungal and corky aroma, and even a trail pheromone. At the same time, pipecholic acid was the only amino acid-based compound found in ramin wood extracts.

Interestingly, several furanoids were extracted from *G. bancanus* wood, especially in polar and semi-polar organic solvents such as methanol, ethyl acetate, and dichloromethane. Furfural, a chemical feedstock formed from the natural dehydration of xylose and arabinose, was found with 2-furfurylthiol and furanone-based compounds. Those furanoid compounds are known as odorous and flavoring agents in food product processing. They represent a solid odor of roasted coffee and its bitter taste, caramel-like aroma, maple syrup flavor, and even an attractive sensory property of strawberry furanone or pineapple ketone (trade name of furaneol).

### Major chemical constituents in *G. bancanus* wood

GCMS analyzes the small molecular weight volatile compounds and compares them according to the mass spectral database defaulted in the instrument. The measurement starts with the sample's vaporization process to be detected in the column and eluted by an inert gas in the chromatography system. LCMS facilitated high boiling point molecules (in liquid form) being analyzed chromatographically without vaporization. This technique covers many compounds predominant as secondary metabolites, even those that are volatile or non-volatile compounds, such as phenolics, saccharides, and complex terpenoids. Consequently, more compounds would be detected and identified in LCMS compared to GCMS. It will influence each compound’s relative amount (percentages) in the total extract. Some compounds may be major when detected using GCMS, but they seem to be minors when analyzed using LCMS. However, the chemical components detected by each instrument will depend on the optimal condition for running the chromatography system.

A case study was raised in this manuscript for linalool. The GCMS data showed that linalool presented in the methanolic extract only, while LCMS showed that linalool was detected in all extracts, except in the methanolic extract. It happened because LCMS detected significant amounts of flavonoids, phenyl propanoids, and saccharides in the methanolic extract. Terpenoids, presented in very small amounts inside methanolic extract, become less detected or could be undetected as a trace when analyzed using LCMS. Whenever the methanolic extract was analyzed using GCMS, the non-volatile compounds, including phenolic and saccharide compounds, became undetected, but the volatile ones, including terpenoids, appeared in significant numbers. On the other hand, the dichloromethane and n-hexane extracts showed less into no trace amounts of phenolics and saccharides when they were analyzed using LCMS. Hence, it caused the terpenoids inside those extracts were detected in considerable amounts. When the dichloromethane and n-hexane extracts were analyzed using GCMS, all the volatile compounds were detected significantly. However, the linalool percentage was assumed to be lower than other volatiles in dichloromethane and n-hexane extracts. It resulted in undetected amount of linalool when the dichloromethane and n-hexane extracts was analyzed using GCMS.

Major chemical components correspondingly correlate to marker compounds of a plant extract. According to Table 4, the major compounds of *G. bancanus* wood are saccharides and flavonoids in methanolic extract; flavonoids in ethyl acetate extracts; terpenoids and flavonoids in dichloromethane extract; terpenoids in n-hexane extract; and flavonoids and terpenoids in the essential oil. Kaempferol is a significant compound in essential oil and all extracts except *n-*hexane extract. It is interesting to discuss when the LCMS data confirmed that kaempferol is a primary compound in the essential oil. Generally, almost no publication on essential oil research states information about the presence of compounds other than terpenoids and aromatics (benzene derivatives) in their essential oil products. It can be understood since essential oil analysis is usually conducted using GCMS. However, the possibility of non-volatile components inside the essential oil was also investigated and analyzed using LCMS in this research. Consequently, the flavonoids, kaempferol, and quercetin, appear as two significant compounds in the essential oil of *G. bancanus* wood. The data compared to GCMS analysis showed that sesquiterpenes are major volatile components in the essential oil. At the same time, isomenthol, linalool, and limonene are three significant compounds in essential oils and all the extracts, except in methanolic extract. Remarkably, linalool is a terpenoid besides the other two terpenoids found only in essential oil of ramin wood, isomenthol, and limonene. However, 10-epi-γ-eudesmol and β-eudesmol are two major compounds comprised of a half composition in essential oil and all extracts.

**Table 4:** Major compounds in the extracts and essential oil of *G. bancanus* based on LCMS and GCMS analysis.

No	Metabolites				
	Methanol	Ethyl acetate	Dichloromethane	*n-*hexane	Essential oil
LCMS					
1	Mannan	Kaempferol	Isomenthol	Isomenthol	Kaempferol
2	Stachyose	Quercetin-3-glucoside-7-rhamnoside	Linalool	Linalool	Isomenthol
3	1(F)-a-D-galactosylraffinose	Quercetin-3-O-malonylglucoside	Limonene	Limonene	Linalool
4	1F-fructofuranosyl nystose	Procyanidin A2	Kaempferol	(2*R*)-Nonan-2-ol	Limonene
5	Kaempferol	Quercetin	(2*R*)-Nonan-2-ol	cis-Rose oxide	Quercetin
GCMS					
1	10-Epi-g-eudesmol	10-Epi- g-eudesmol	10-Epi- g-eudesmol	10-Epi- g-eudesmol	10-Epi- g-eudesmol
2	b-Eudesmol	b-Eudesmol	b-Eudesmol	b-Eudesmol	b-Eudesmol
3	1-Methyl-4-(2-methyloxiranyl)-7-oxabicyclo 4.1.0 heptane	1-Methyl-4-(2-methyloxiranyl)-7-oxabicyclo 4.1.0 heptane	1-Methyl-4-(2-methyloxiranyl)-7-oxabicyclo 4.1.0 heptane	Oleic acid	Dihydrocarvyl acetate
4	6-Methyl-5-heptene-2-one	3,3,6,6,9,9-Hexamethyl-tetra cyclo [6.1.0.0(2,4). 0(5,7)] nonane	Oleic acid	Squalene	6-Methyl-5-heptene-2-one
5	Limonene dioxide 1	Oleic acid	a-Copaene-11-ol	Dihydrocarvyl acetate	a-terpineol

The presence of eudesmol in ramin wood is interesting to discuss since this bicyclic sesquiterpenol has been a marker compound for essential oil from ramin wood, gaharu bouya. The three isomers of eudesmol: alpha, beta and gamma, smell mildly sweet and primarily woody. β-eudesmol shows many reported biological activities, including antidote for intoxication^105^, neuromuscular blockade^106^, antiepileptic action^107^, anti-leaf-cutting ant^108^, antimicrobial activity^109^, antitumor and anti-angiogenic activities^110^, and stimulating appetite^111^. While 10-epi-γ-eudesmol is a potential repellent against *Aedes aegypti* (L.)^112^ and *Amblyomma Americanum* (L.) nymphs^113^ and is firmly attached to anti-inflammatory and immunomodulatory receptors^114^. This compound, 10-epi-γ-eudesmol, has also been found to be a featured or a marker compound for high-quality agarwood (gaharu) oil from *Aquilaria* genus^16,115–121^.

### The influence of solvent polarity on the identified compound composition of *G. bancanus* wood

To comprehensively characterize the metabolite fractions of *G. bancanus* wood, the whole part of wood was extracted using various solvents in a range of polarity, from *n-*hexane (non-polar) to methanol (polar). Partitioning of metabolites between different extraction solvents is important and could provide comprehensive coverage of ramin wood metabolome. Commonly, metabolites are detected in one solvent fraction only, in which case better quantitation with higher detection sensitivity and more efficient data analysis can be achieved. Based on polarity, a metabolite could be extracted by a solvent that can be fully dissolved, as a principle of like dissolves. However, single use of extraction solvent may provide fewer countable metabolites in the sample compared to a series of different polarity of solvent extractions. Although each separated fraction can provide complementary information, total extraction using different polarity solvents could lead to broader metabolite coverage. Therefore, a range of solvents in different polarities was appropriate for this metabolic profiling.

PCA is used for data visualization and data classification. As shown in Fig. 2a, the methanolic fraction of ramin wood contained a higher amount of highly polar, including flavonoids, saccharides, and phenyl propanoids. The ethyl acetate fraction had fewer saccharides but a bulk of flavonoids and terpenoids. Dichloromethane fractions collected terpenoids, flavonoids, and miscellaneous such as (2*R*)-nonan-2-ol and 3-hexenyl acetate, a fresh fruity flavor and fragrance agents. Interestingly, essential oil as a distillation product contains terpenoids, which are known as a volatile compound class, and non-volatile compounds such as flavonoids and phenyl propanoids. This fact commonly causes an essential oil to have an antioxidative property, especially in its components' presence of polyhydroxyl groups.

Furthermore, compared to LCMS data, GCMS analysis exhibited a uniform pattern of compound class from ramin wood extracts and essential oil. Detected components in ramin wood extracts mostly contain more than 50% oxygenated sesquiterpenes. Therefore, according to this discussion, extraction using methanol and *n-*hexane is suggested to apply for a comprehensive metabolomic of ramin wood.

To strengthen the previous discussion of the solvent effect and to analyze the interrelations between the extracts, the LCMS data were submitted to multivariate analysis by PCA (Fig. 1a). The relative amount of each identified compound from each extract was considered a variable, and each extract's score was calculated. The PC1 accounted for 66.26%, and the PC2 for 20.75% of the total variation in the dataset. It is possible to distinguish two clusters of extract and to highlight the differences in the PCs profile between the extraction solvents. PC1 axis helped separate the components of essential oil and dichloromethane-hexane extracts from methanol and ethyl acetate extracts. It was marked by a high concentration of geranial in essential oil; other terpenoids inside the essential oil and *n-*hexane extract; aromatics, and miscellaneous in the dichloromethane and *n-*hexane extracts.

Conversely, extracts with a low score on PC1 gathered methanol extract marked with organic acids, ethyl acetate extracts with alkaloids, and a combination of methanol and ethyl acetate with flavonoids. PC2 resumed less variability, with the most distancing to the centroid (Fig. 1c) is the hydrodistillation product (essential oil) on the superior quadrant with high scores. The implications of this observation are significant for determining which solvent is suitable for extracting all the metabolites in *G. bancanus* wood. Two solvent options could be selected for each available cluster (Fig. 1c) to facilitate a comprehensive metabolomic of ramin wood. An example of this is applying *n-*hexane as a representative solvent of the first cluster (C1), and methanol as a representative from the second cluster (C2) for metabolic profiling of ramin wood.

According to the PCA biplot of GCMS data in Fig. 1b, identified compounds in *n-*hexane extract were separated from others. Terpenoids were distributed evenly among extracts, but it was clearly shown as responsible compounds for separation, including longipinenepoxide in the superior quadrant and 6-methyl-5-hepten-2-one in medium one, while α-bisabololoxide B and α-terpineol in inferior quadrant. By applying an unsupervised multivariate analysis, no clear variance separation on the negative quadrant of PC1 can be observed (Fig. 1b). PCA could separate *n-*hexane and dichloromethane extracts from other extracts. Still, the rest of it, including essential oil, ethyl acetate, and methanol extracts, are hard to discriminate since the distance of these three extracts in the negative quadrant of PC1 is very close, meaning the level concentration of the 89 peak compounds was not so high in the three extracts. Therefore, performing a three-dimensional analysis on GCMS data (Fig. 1e) helped separate variance clearly in three clusters corresponding to AHC in Fig. 1d. According to the comparison of the extraction potentials among different polarities of extraction solvents using GCMS analysis, *n-*hexane is a potential solvent to produce a discriminate chemical constituent from others.

## Conclusion

Metabolite profiling of *G. bancanus* wood has been performed from a series of polarity solvent extractions using methanol, ethyl acetate, dichloromethane, *n-*hexane, and hydrodistilled-essential oil. The 142 identified metabolites from LCMS and 89 metabolites from GCMS revealed a wide range of compound classes: terpenoids, phenolic compounds (flavonoids and phenyl propanoids), and saccharides. Major terpenoids in *G. bancanus* wood extracts are isomenthol, linalool, limonene, 10-epi-g-eudesmol and b-eudesmol. Kaempferol and quercetin glycosides are the main flavonoids found in ramin wood. Saccharides such as mannan and sucrose derivatives, including stachyose, raffinose, and nystose, are also bulk in methanolic extract. PCA biplots of two kinds of analysis (LCMS and GCMS) demonstrated discrimination of components when *n-*hexane was applied to the extraction. Combining *n-*hexane with another solvent, such as methanol, could help a comprehensive extraction since it resulted in different components from *n-*hexane. This research result validates the use of *n-*hexane and another cluster of ethyl acetate, methanol, and hydrodistillation (a choice) to provide broad coverage of ramin wood metabolomics.

## Materials and methods

### Chemicals

Methanol, ethyl acetate, dichloromethane (methylene chloride), and *n-*hexane were analytical grades from Merck and Fulltime chemical suppliers.

### Plant materials

**Figure 3:**
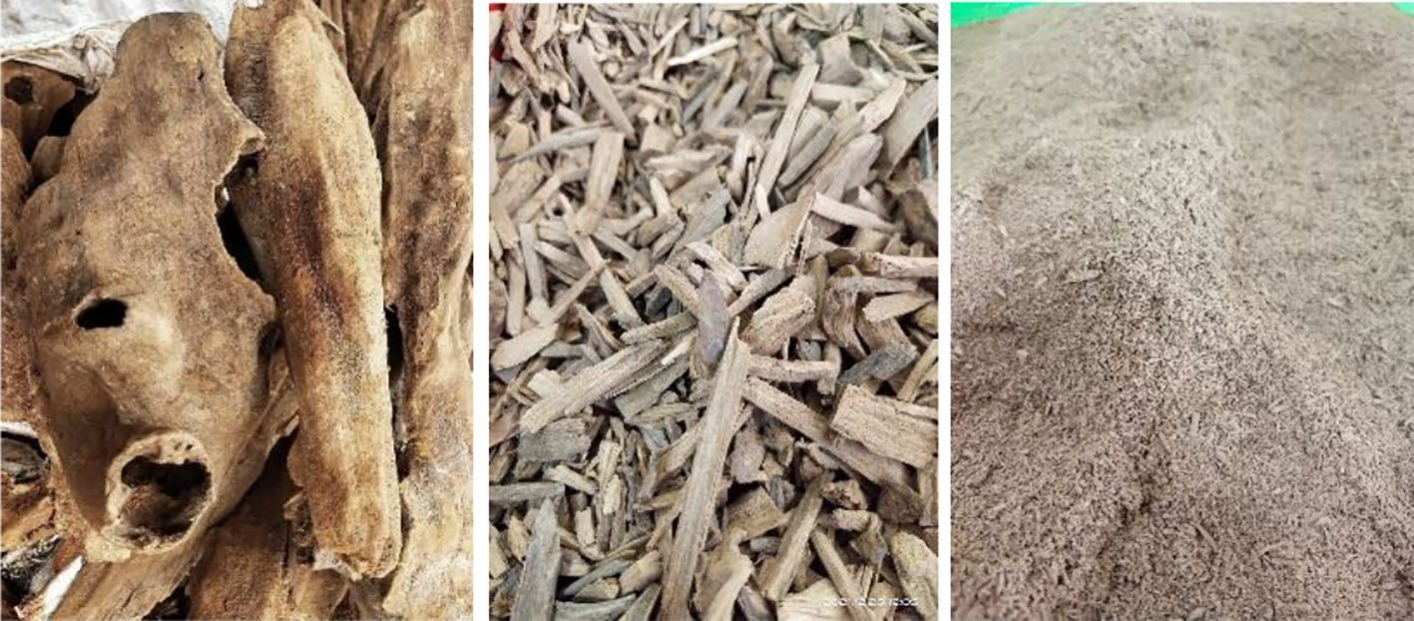
The physical appearance of the sample. From left to right: wood trunk, wood chips, and wood powder of *G. bancanus*.

The wood of *Gonystylus bancanus* was obtained initially from the forest of Middle Kalimantan, Indonesia, and has entered the market in East Java, particularly in Balung, Jember district as a legal log. The wood chips were distributed legally by the essential oil producer and exporter of PT Padaelo Sejahtera, Magelang, Central Java, Indonesia. The wood was formally identified by wood anatomists, Prof. Agus Budi Sulistiyo and Ms. Sri Wahyuni, at Laboratory of Biology and Wood Preservation, Faculty of Forestry, Universitas Mulawarman, Indonesia. The specimen of *G. bancanus* was deposited in this laboratory with voucher number 220622-3. The experimental field study complies with relevant institutional guidelines and is carried out in accordance with relevant regulations. The wood chips were grounded into powder (Fig. 3) using a Fomac mill processor. The moisture content of the wood powder was directly determined after grinding. The samples of wood powder were kept in a dry box before extraction.

### Solvent extraction

A hundred grams of *G. bancanus* wood powder was put on four erlenmeyers. Each erlenmeyer was filled with different solvents (± 200 mL): methanol, ethyl acetate, methylene chloride, and n-hexane, until it covered all of the surfaces of the powder. After 48 h of maceration, the mixture was filtered to obtain a filtrate (extract) and residue (of wood powder). The residue was remixed with the same solvent and was macerated for two days. The procedure was repeated three times, and the extracts were collected in a big flask. The extract was then evaporated under reduced pressure using a rotary evaporator to obtain different forms of crude extracts, *i.e.,* a dark red wet powder of methanol extract, a dark yellow to thick black oil of ethyl acetate extract, a dark orange viscous oil of methylene chloride extract; and a light-yellow oil of *n-*hexane extract. Those four extracts were subjected to analysis using GCMS and LCMS.

### Distillation

Five hundred grams of wood powder was distilled using a Clevenger hydrodistillation set-up with an addition of 3.5 L aquadest. Distillation was run for 6 h and was counted for the first second at the first drop of distillate. The essential oil in the distillate was separated from the water and was dried using anhydrous magnesium sulfate. Resinous oil was obtained and directly sent for further analysis using GCMS and LCMS.

### Liquid chromatography mass spectrometry analysis

LCMS analysis was performed on a Shimadzu LCMS-8040 LC/MS equipped with a Shimadzu Shim Pack FC-ODS column of 2 mm × 150 mm, and 3 µm. The LCMS properties comprised an injection volume of 1 µL, a capillary voltage of 3.0 kV, and a column temperature of 35 °C. Mobile phase mode was isocratic with a 0.5 mL/min flow rate and a sampling cone of 23,0 V. The MS-focused ion mode was ion type [M]^+^ with a collision energy of 5.0 V, desolvation gas flow of 60 mL/h, and desolvation temperature of 350 °C. The fragmentation method was low energy CID with ionization by ESI, scanning rate was 0.6 sec/scan (m/z: 10–1500), source temperature of 100 °C, and run time of 80 min.

### Gas chromatography-mass spectrometry analysis

GCMS analysis was run on Shimadzu GCMS-QP2010S equipped with DB-5MS column in 30 m length, 0.25 mm diameter, and 0.25 μm wide of film. The carrier gas was Helium, with ionization of EI 70 eV. The column oven temperature was 70.0 °C held for 5.00 min, while the injection temperature was 300.00 °C for 19.00 min. Injection mode was split with flow control mode was pressure at 15.5 kPa. The total flow was 28.8 mL/min, column flow was 0.52 mL/min, linear velocity was 26.3 cm/s, purge flow was 3.0 mL/min, and split ratio was 49.0. The ion source temperature for MS was 250.00 °C, the interface temperature was 305.00 °C with a solvent cut time of 3.00 min, and the detector gain mode was relatively + 0.00 kV.

Spectrums and their fragmentations obtained from LCMS and GCMS analysis were matched to the spectrum references under the Mass Spectral Library of NIST20 and WILEY229—NIST62 databases, respectively. The instruments are regularly standardized using a reference mass of perfluorotributylamine (PFTBA, C_12_F_27_N) and PEG-PPG-Raffinose, respectively. These databases confirm a range of volatile and non-volatile compounds.

### Statistical analysis

The diagnostic tool for statistical analysis in this research included of score plot and loading plot (shown in the biplot figure) of Principal Component Analysis (PCA), a dendrogram of Hierarchical Clustering Analysis (HCA), and a 3D plot of Origin software. Biplot analysis was performed on data of LCMS and GCMS analysis as a graph of extraction solvents toward the relative amount of correlated identified compounds. The clustering of the extracts was determined from the identified compounds resulting from the variation of solvent extraction and was mapped on HCA. Whenever the accumulative eigenvalue of PCs was less than 80.0%, an increasing matrix was compulsory to apply until the minimum PC value of 80.0% was obtained. Origin software with a three-dimension plot was preferred to explain this insufficient eigenvalue.

## Data Availability

All data generated and analyzed during this study are included in this paper. Further detail for those related data are available from the corresponding author on reasonable request.
